# Thermogenic capacity of human periaortic adipose tissue is transformed by body weight

**DOI:** 10.1371/journal.pone.0194269

**Published:** 2018-03-19

**Authors:** Diana Vargas, Carolina López, Edward Acero, Edgar Benitez, Angélica Wintaco, Jaime Camacho, Marisol Carreño, Juan Umaña, Daniela Jimenez, Said Díaz, Fernando Lizcano

**Affiliations:** 1 Center of Biomedical Investigation from Universidad de La Sabana (CIBUS), Chía, Cundinamarca, Colombia; 2 Fundacion CardioInfantil-Instituto de Cardiología, Bogotá, D.C., Colombia; State University of Rio de Janeiro, BRAZIL

## Abstract

The anatomical location of adipose tissue might have direct implications for its functionality and risk of cardiovascular disease. Adipose tissue surrounding blood vessels may be thermogenically more active in specific areas of the body, releasing substances that regulate vascular metabolism. In humans, the phenotypic characteristics of adipose tissue surrounding the aorta and the cardiovascular disease risk that it might entail remain largely unknown. Here, we compared thermogenesis-related molecular features of human periaortic adipose tissue samples with those of subcutaneous adipose tissue, obtained by sternotomy from 42 patients undergoing cardiovascular surgery. To determine the expression of genes related to energy expenditure and the levels of some adipokines, histological examinations, quantitative PCR, and protein expression measurements in adipocyte precursor cells were performed. Periaortic adipocytes were smaller than those from subcutaneous tissue. Moreover, weight gain induced periaortic adipocyte hypertrophy (r = -0.91, p<0.01). Compared to subcutaneous tissue, adiponectin, FABP4, IL-4 and IL-6 was decreased in periaortic adipocytes, whereas FGF21, UCP-1, PGC-1a, CITED1, Omentin and TFAM (Mitochondrial protein) increased. Upon analyzing patients’ clinical conditions, it emerged that the levels of PGC-1a both in male (r = -0.48 p<0.04) and female (r = -0.61, p<0.05) and TFAM in male (r = -0.72, p<0.0008) and female (r = -0.86, p<0.002) decreased significantly with progressive weight gain. However, no differences were observed in patients with diabetes mellitus 2 or Hyperlipidemia. Adipocytes surrounding the ascending aorta present markers of major thermogenic activity than those in subcutaneous tissue. Nevertheless, this characteristic might change, due to unfavorable metabolic conditions such as obesity, which is a risk factor for cardiovascular disease.

## Introduction

Obesity has become a global public health problem and contributes to the development of cardiovascular disease [1, 2]. Its high incidence prompted a detailed assessment of the local effect exerted by adipose tissue in relation to its anatomical location. Recently, the importance of adipose tissue surrounding blood vessels, known as perivascular adipose tissue (PVAT), has been revealed. Its expansion and associated inflammation are involved in the development of endothelial dysfunction and atherosclerosis as well as in the pathogenesis of insulin resistance and metabolic syndrome [3–6]. PVAT can exert endocrine and paracrine actions, by secreting a wide range of bioactive molecules that influence vascular metabolism [7, 8]. Studies in animal models of obesity showed that PVAT could change its vasoregulatory capacity in view of a lower release of vasodilator adipokines and a higher release of additional factors promoting vasoconstriction [7, 9]. In contrast, adipose tissue might modulate physiology in terms of its energy capacity. The ability to oxidize fatty acids without causing their accumulation in white adipocytes, allows brown adipose tissue (BAT) to reduce adiposity [10]. In humans, the most thermogenically active adipose tissue is brown-like or beige. It exhibits the same metabolic properties as BAT, although with different precursors [11]. This thermogenically active phenotype is important in a vascular context, because mouse periaortic adipose tissue (PAT) located at thoracic level, presents brown-like features [12]. In humans, thermogenesis-related proteins are expressed by the adipose tissue surrounding coronary arteries. Previously we showed that capillary vascularization is higher in PAT than in sWAT, indicating that it has increased metabolic activity [13, 14]. It is known that different vascular niches can present different phenotypic characteristics, depending on their anatomical location [15]. The metabolic relevance of brown and beige adipose tissue is highlighted by studies in which its protective effect has been shown to go beyond its ability to oxidize fatty acids. These include changes in lipid metabolism, such as reduced levels of cholesterol, low-density lipoprotein (LDL), and very low-density lipoprotein, which are known to counteract atherosclerosis in hyperlipidemic mice [16]. Studies evaluating the potential risk of cardiovascular disease induced by PAT and based on imaging records, reported that weight gain was related to an increase in PAT [4]. The Framingham heart study observed high PAT measured by computed tomography associated to an increase in hepatocyte growth factor in women [17]. Nevertheless, energy activity and release of adipokines by PAT in humans remains poorly characterized.

The objective of this study was to phenotypically and functionally characterize adipose tissue located in the ascending aorta (PAT) and obtained from patients subjected to revascularization or mitral valvular replacement. Samples of subcutaneous adipose tissue (sWAT) were obtained from the same patient for comparative purposes. Morphological examination showed that adipocytes were smaller in PAT than in sWAT, in a manner that depended on body mass index (BMI). RNA expression analysis indicated reduced adiponectin and fatty acid binding protein 4 (FABP4) levels in PAT, whereas immunohistochemistry showed increased levels of fibroblast growth factor 21 (FGF21). Protein expression analysis indicated that PAT exhibited a more active thermogenic behavior compared to sWAT, due to a significantly higher expression of PPARg coactivator 1 alpha (PGC-1a). Interestingly, thermogenic capacity of periaortic fat decrease with an increase in the Body fat % estimation, at least observing the reduction of the expression of PGC-1a and the transcription factor A, mitochondrial (TFAM), possibly indicating that in conditions such as obesity, the physiology of PAT might change, and the risk of cardiovascular disease might increase. We did not observe these characteristic changes in patients with diabetes mellitus 2 or dyslipidemia.

## Materials and methods

### Patients characterization

The present study included 42 patients, of whom 82.4% had coronary artery disease and 67% were males. The average age was 62±9 years for males and 63±9 years for females. Most patients (55.9%) presented a BMI of 25–30 kg/m^2^ indicating that they were overweight, whereas 20.6% were obese (>30 kg/m^2^). Among women, 45.4% had normal BMI, 36.4% were overweight, and 18.2% were obese. Among men, 21.7% had normal BMI, 56.6% were overweight, and 21.7% were obese. Body fat % estimation, was performed trough Clínica Universidad de Navarra-Body Adiposity Estimator (CUN-BAE) method and was calculated as −44.988+ (0.503 x age) + (10.689 x sex) + (3.172 x BMI) − (0.026 x BMI^2^) + (0.181 x BMI x sex) − (0.02 x BMI x age)–(0.005 x BMI^2^ x sex)+ (0.00021 x BMI^2^ x age) where male = 0 and female = 1 for sex, and age in years[18, 19]. Additionally, 14.7% of patients had hypothyroidism (TSH >5.4 mU/L). Finally, 29.4% of patients had LDL levels above 150 mg/dL, 47% had levels of triglycerides above 150 mg/dL, 29.4% were diabetic, 20.6% presented glycated hemoglobin HbA1c in the pre-diabetes range, and 50% in the normal range (Table 1).

**Table 1:** Clinical characteristic from 42 patients included in the study. 26 samples were taken for western blot analysis, 8 samples for immunohistochemistry and 8 for RT-PCR.

		No. of Patients
**Female/Male**		14/28*
**Age (years)**		**62±8**
**BMI (kg/m2) Normal**	**18–24.9 (kg/m2)**	**10**
**BMI (kg/m2) Overweight**	**25–29.9 (kg/m2)**	**22**
**BMI (kg/m2) Obese**	**>30 (kg/m2)**	**10**
**DM 2**	**A1c > 6.2%**	**9**
**Pre-DM2**	**A1c (5.7–6.2%)**	**8**
**Hypothyroidism**	**TSH > 5.4 mU/L**	**6**
**Hypercholesterolemia**	**Cholesterol LDL> 150**	**13**
**Hypertriglyceridemia**	**Triglycerides >150**	**22**
**Coronary disease**	**Myocardial revascularization**	**33**
**HTA**	**Systolic/diastolic Blood Pressure > 140/90**	**29**

*Relation between Female and Male from patients that participate in the study.

### Tissue collection

Patients were subjected to elective cardiovascular surgery for myocardial revascularization or mitral valve surgery at the Fundacion CardioInfantil-Instituto de Cardiología. Samples were identified and obtained from PAT at the level of the ascending aorta and from subcutaneous adipose tissue in the sternotomy area. Before surgery, participants reviewed and signed the informed consent form. The study was approved by the Ethics Committee of the University La Sabana and Cardioinfantil Foundation (Bogotá D.C., Colombia). Because the limited amount of tissue sample obtained from PAT, some samples were only examined for histological analysis. The distribution of the samples was carried out randomly for the experiments with RNA, immunohistochemical and western blot. Out of a total of 74 samples obtained from December 2015 to May 2017, only 42 samples of periaortic tissue were viable. Because the objective of the work was the study of the expression of proteins, most of the samples were used for the realization of western blot.

### Histological analysis and immunohistochemical tests

sWAT and PAT samples were fixed in 10% formaldehyde in phosphate-buffered saline (PBS), pH 7.4, at room temperature. After fixation, tissues were dehydrated in ethanol and then washed in Xylol, embedded in paraffin, and cut into 4-μm sections using a microtome (American Optical). Analysis of adipocytes was conducted using Zen lite software on 40× images, comprising four different fields per sample. Samples were analyzed, tracing manually the outline of the adipocytes found in each field (Carl Zeiss AXIO observer A1 equipped with an AxioCam ICc1, Jena, Germany). Morphological analysis was performed by the tests system named M42 (multipurpose test-system) [20] that has 42 test-points. Volume densities (Vv) of adipocytes were determined by point counting, Vv (volume densities: Vv = Pp/Pt%, Pp is the number of test points in the structure, Pt—number of total test points. This stereological test was mounted in microscopy Zeis Axio observer (Carl Zeiss, Jena, Germany) [21] in sWAT and PAT in samples from eight patients, who presented a BMI of 26.7±2 kg/m^2^. Subsequently, tissue sections from PAT and sWAT were incubated with anti-FGF21 (1:500) and anti-omentin (1:200) antibodies. Samples were then incubated with a secondary rabbit anti-IgG antibody (Novocastra, Newcastle Upon Tyne, United Kingdom; 1:200) for 30 min. Developing was carried out with a conventional peroxidase technique using 3',3'-diaminobenzidine hydrochloride chromogen (Sigma, St. Louis, MO) following a 20-min incubation. This was then followed by three washes with PBS at 10-min intervals. Subsequently and to provide contrast, the immunostained slides were stained with a 33% hematoxylin solution for 20 s and washed with PBS for 3 min. The slides were visualized as specified above. Immunostaining was considered positive when the characteristic brown color was observed according to the protein identified in the tissue. Relative antibody signal intensity was quantified using Image J software.

### Quantitative PCR

Adipose tissue samples (sWAT and PAT) were collected and transported in liquid nitrogen for subsequent RNA extraction. Starting with 90 mg of adipose tissue, disruption was performed with liquid nitrogen and samples were homogenized with Lysis Buffer using the High Pure RNA Isolation Kit (Roche Diagnostics, Mannheim, Germany) according to the manufacturer's instructions. Then, 100 ng of RNA was used to generate cDNA using the Transcriptor First Strand cDNA Synthesis Kit (Roche Diagnostics). Quantitative PCR reactions were carried out to detect expression of adiponectin (Fw 5′-TTCACCGATGTCTCCCTTAGG-3′ and Rev 5′-GGCATGACCAGGAAACCAC-3′), FABP4 (Fw 5′-AGCACCATAACCTTAGATGGGG-3′ and Rev 5′-CGTGGAAGTGACGCCTTTCA-3′), PGC-1a (Fw 5′-CTGTGTCACCACCCAAATCCTTAT-3′ and Rev 5′-TGTGTCGAGAAAAGGACCTTGA-3′), IL-4 (Fw 5’- GTGCTATGTCAGCATCACCAAGA 3', Rev: 5' CCCCTGAGCATCCTGGATTAT3'), IL-6 (Fw 5’- AGTTGCCTTCTTGGGACTGA3', Rev: 5' TCCACGATTTCCCAGAGAAC3'), UCP-1 (Fw 5′-GTGTGCCCAACTGTGCAATG-3′ and Rev 5′-CCAGGATCCAAGTCGCAAGA-3′), following instructions on the FastStart Essential DNA Green Master (Roche Diagnostics). For quantitative analysis, mRNA expression was normalized using glyceraldehyde 3-phosphate dehydrogenase (GAPDH) (Fw 5′-ACCCACTCCTCCACCTTTGAC-3′ and Rev 5′-TGTTGCTGTAGCCAAATTCGTT-3′) and the ΔΔCt method.

### Cell cultures

Adipose tissue samples were washed with PBS and digested with 250 U/mL type I collagenase, 20 mg/mL bovine serum albumin, and 60 ug/mL gentamycin in PBS for 60 min at 37°C with agitation. Next, samples were centrifuged for 5 min at 200 × *g* and the pellet was resuspended in an erythrocyte lysis buffer composed of 154 mM NH_4_Cl, 5.7 mM K_2_HPO_4_, and 0.1 mM EDTA pH 7.3 for 10 min. The mixture was filtered through a nylon mesh with 150-μm pore size, followed by centrifugation at 200 × *g* for 10 min. The cell pellet was resuspended in proliferation medium (Dulbecco’s modified Eagle’s medium/F12) supplemented with 15% fetal bovine serum and 50 ug/mL gentamycin. After 24 h, cells were washed and incubated again in the same medium supplemented with 10% fetal bovine serum and 50 ug/mL gentamycin, until they reached confluence.

### Protein expression analysis by western blotting

Total proteins were extracted with RIPA buffer (ab156034; Abcam, Cambridge, MA) and 1 ug of protease inhibitors (04693159001; Roche Diagnostics). Samples were quantified using the Bradford method, to obtain a working concentration of 50 ug/ul. Samples were denatured at 95°C, run on a polyacrylamide gel, and transferred to a polyvinylidene fluoride membrane pre-treated with 100% methanol for 2 min. The membrane was blocked with PBS-T (1× PBS and 0.1% Tween-20) and 5% skim milk powder, and then incubated with the following rabbit antibodies: anti-PGC-1α (1:1.000, ab54481; Abcam, Cambridge, MA), anti-TFAM (1:1,000, Ab155117; Cell Signaling Technology, Beverly, MA), and anti-UCP-1 (1:1.000, ab155117; Abcam, Cambridge, MA). A rabbit anti-IgG-HRP secondary antibody was used at a dilution of 1:5.000. Adipokines were detected with the following rabbit antibodies: anti-adiponectin (1:3.000, ab92501; Abcam, Cambridge, MA), anti-FGF21 (1:2.000, ab171941; Abcam, Cambridge, MA), and anti-FABP4 (1:3.000, ab92501; Abcam, Cambridge, MA), anti-IL-6 (1:1.000, 8940, Cell Signaling, Beverly, MA); anti-IL-4 (1:1.000, D19A10, Cell Signaling, Beverly, MA), anti-CITED1 (1:3.000, ab87978; Abcam, Cambridge, MA) and then a secondary rabbit antibody as described above. Detection was performed by chemiluminescence following the Luminata crescendo kit instructions (Millipore, Billerica, MA). Images were captured and analyzed by densitometry using the myECL Imager (Thermo Fisher Scientific, Waltham, MA). Quantitative analyses were performed based on three independent experiments.

### Statistical analysis

The samples showed a normal distribution. The studied variables of interest, e.g., volume density, adiponectin and FABP4 mRNA expression, as well as the relative expression of PGC-1a and TFAM in sWAT and PAT were analyzed using a paired *t*-test. The relationship between BMI and volume density, and between Body fat % estimator (CUN-BAE) and expression of thermogenic proteins (PGC-1a and TFAM) was analyzed using Pearson’s correlation coefficient. Data were expressed as means ± standard deviation (SD), and a p<0.05 was considered statistically significant.

## Results

### Morphological analysis of sWAT and PAT

**Fig 1:**
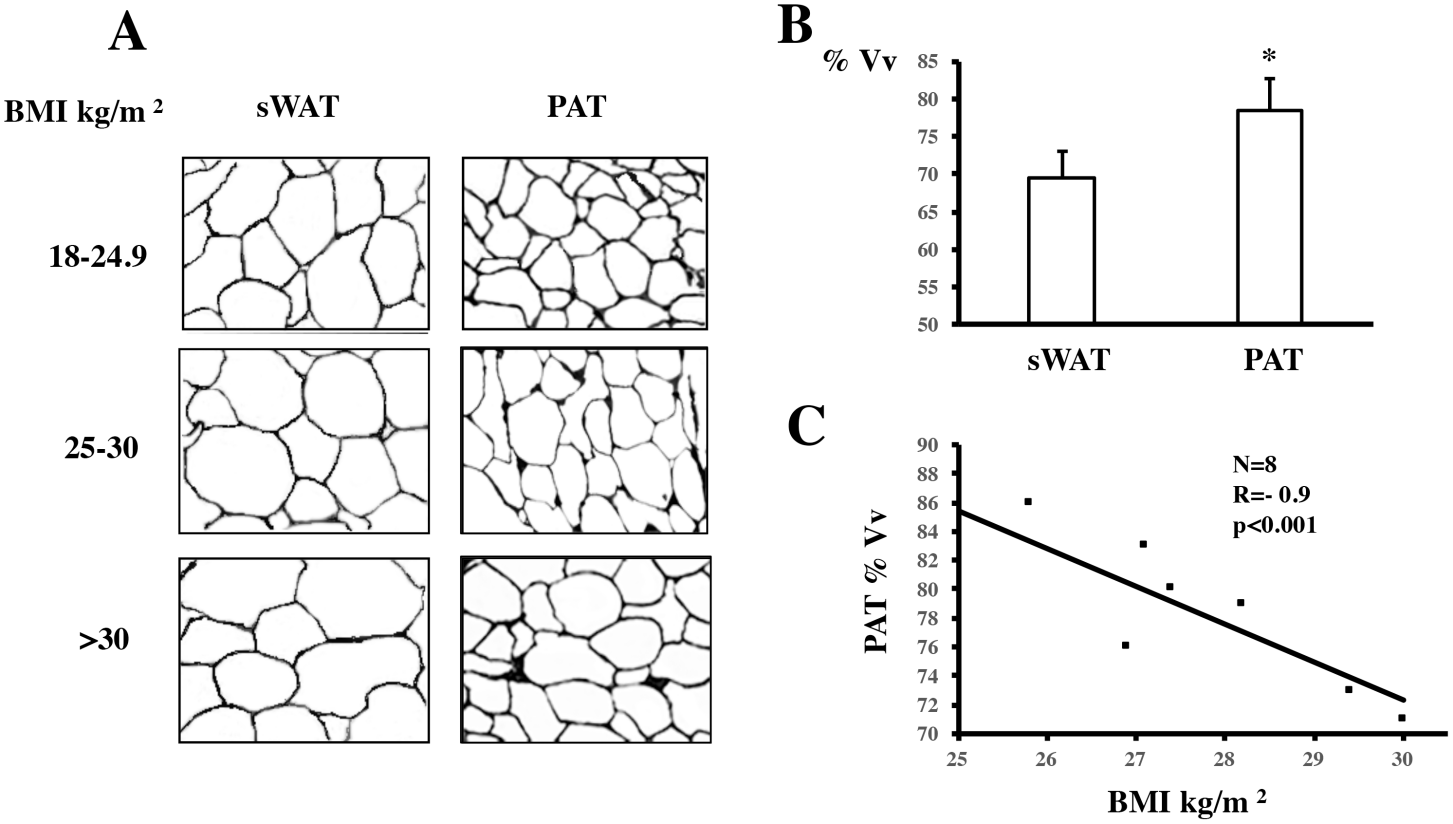
Morphology of subcutaneous adipose tissue (sWAT) and periaortic adipose tissue (PAT) adipocytes. (A) Representative images of formaldehyde-fixed sWAT and PAT samples obtained from patients with normal (18–24.9 kg/m^2^), overweight (25–29.9 kg/m^2^), and obese (>30 kg/m^2^) body mass index (BMI). (B) Quantification of volume density was performed on the captured images, which were performed by M42 (multipurpose test-system) The percentage of adipocyte volume densities were performed and (*) p<0.05 indicates a statistically significant difference between sWAT and PAT adipocyte volume density. (C) Correlation between BMI and adipocyte volume density in PAT as calculated with Pearson’s correlation (r = -0.91, p<0.01). Data are expressed as mean ± SD (n = 8). A paired t-test was performed to determine differences between averages.

The morphological analysis was performed by the tests system named M42 that has 42 test-points, the test-line measures 21d and the test-area measures 36.36d^2^, this sterological test was mounted in microscopy Zeis Axio observer. sWAT and PAT was assessed in samples from eight patients, who presented a BMI of 26.7±2 kg/m^2^. Microscopic observations showed that, volume density (Vv) was significantly higher in the PAT in relations with sWAT adipocyte (81.2±5.2% vs. 68.3±4.1%, p<0.05) Fig 1A and 1B. Given a possible relationship between adipocyte volume density and BMI in PAT, we performed a Pearson’ correlation analysis with the BMI in eight patients in three different fields. Results showed a negative correlation between adipocyte Vv and the increase in BMI (r = -0.92, p = 0.001) in PAT, Fig 1C. When BMI increased, the difference in adipocyte area between sWAT and PAT samples became smaller. The volume density of PAT adipocytes was significantly reduced with the increase of the BMI, signifying the development of a hypertrophy process in the adipose cells. Thus, PAT was seen to lose its characteristic phenotype with increasing weight.

### Gene expression in sWAT and PAT

**Fig 2:**
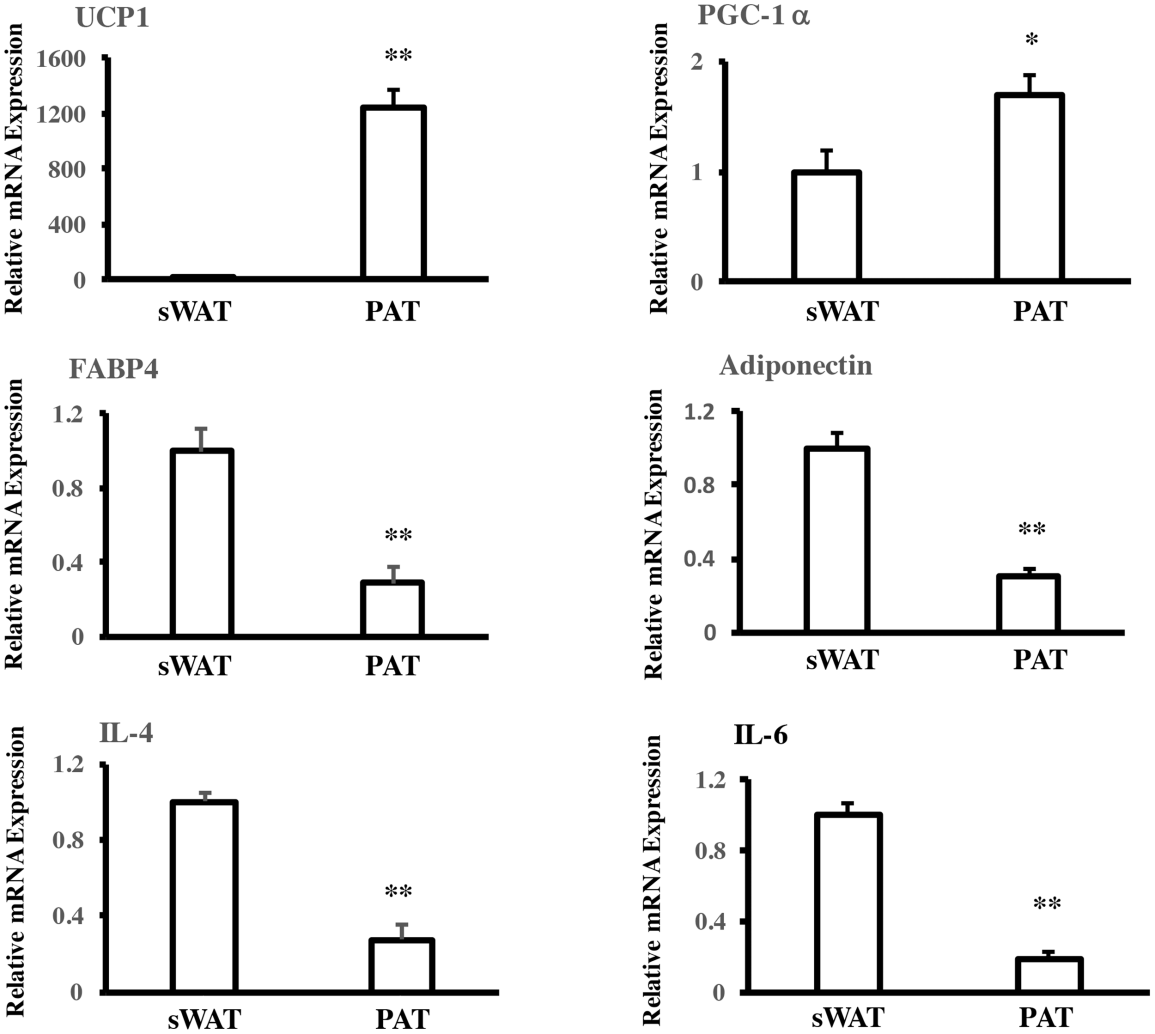
Adipokine and thermogenic mRNA expression in subcutaneous adipose tissue (sWAT) and periaortic adipose tissue (PAT). Quantitative PCR analysis of sWAT and PAT samples showing the expression of some thermogenic, adipokine and inmmune genes. UCP-1, Uncoupling protein 1; PGC-1a, PPARg coactivator 1 alpha; FABP4, fatty acid binding protein 4; IL-4, Interleukin 4; IL-6, Interleukin 6. Quantitative PCR analysis showing the expression of * p<0.05 and ** p<0.01 (n = 8) indicate a statistically significant difference between gene expression levels in sWAT and PAT (mean ± SD).

To explore possible phenotypic differences between sWAT and PAT, the expression of some adipokines genes were assessed. Samples from some patients where a good volume of tissue was obtained were induced to differentiation. The adipocyte Precursors were induced to differentiation with the cocktail of differentiation for 10 days. Expression of adiponectin, FABP4 and IL-4 and IL-6 mRNA was determined in paired samples from patients with a BMI of 27.3±2.1 kg/m^2^. These genes were significantly more expressed in sWAT than in PAT. In contrast, expression of thermogenic genes PGC-1a and uncoupling protein 1 (UCP-1) was significantly higher in PAT, Fig 2.

**Fig 3:**
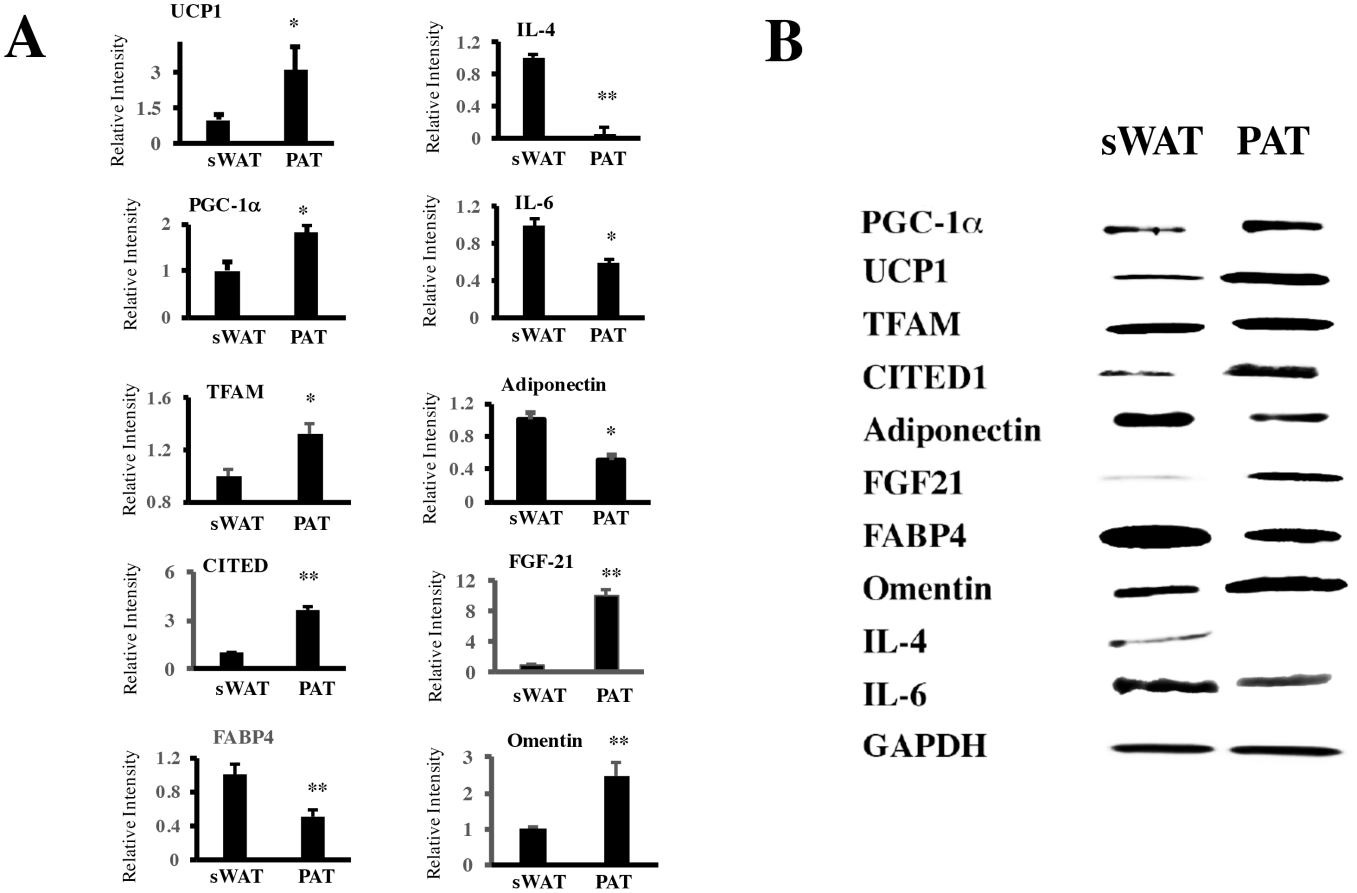
Protein expression of in subcutaneous adipose tissue (sWAT) and periaortic adipose tissue (PAT). (A) Western Blot was performed in patients with BMI normal (18–24.9) to compare the expression of different proteins. UCP-1, Uncoupling protein 1; PGC-1a PPARg coactivator 1 alpha; FABP4, fatty acid binding protein 4; IL-4, Interleukin 4; IL-6, Interleukin 6; TFAM, transcriptor factor A, mitochondrial precursor; CITED1, CBP/p300 interactive transactivator 1; FGF21, fibroblast growth factor 21. * p<0.05 and ** p< 0,01 mean ± standard deviation (SD) of relative density of antibodies between of sWAT and PAT. In (B), a representative image of Western blot.

The level of protein expression confirmed the observations in mRNA, with an increase in some thermogenic proteins CITED1, PGC-1a and TFAM. With a reduction in adipogenic and inflammatory proteins FABP4, Adiponectin, IL-4 and IL-6. FGF21 and Omentin, proteins that have a favorable cardiovascular function, were observed augmented in PAT adipoctyes Fig 3.

**Fig 4:**
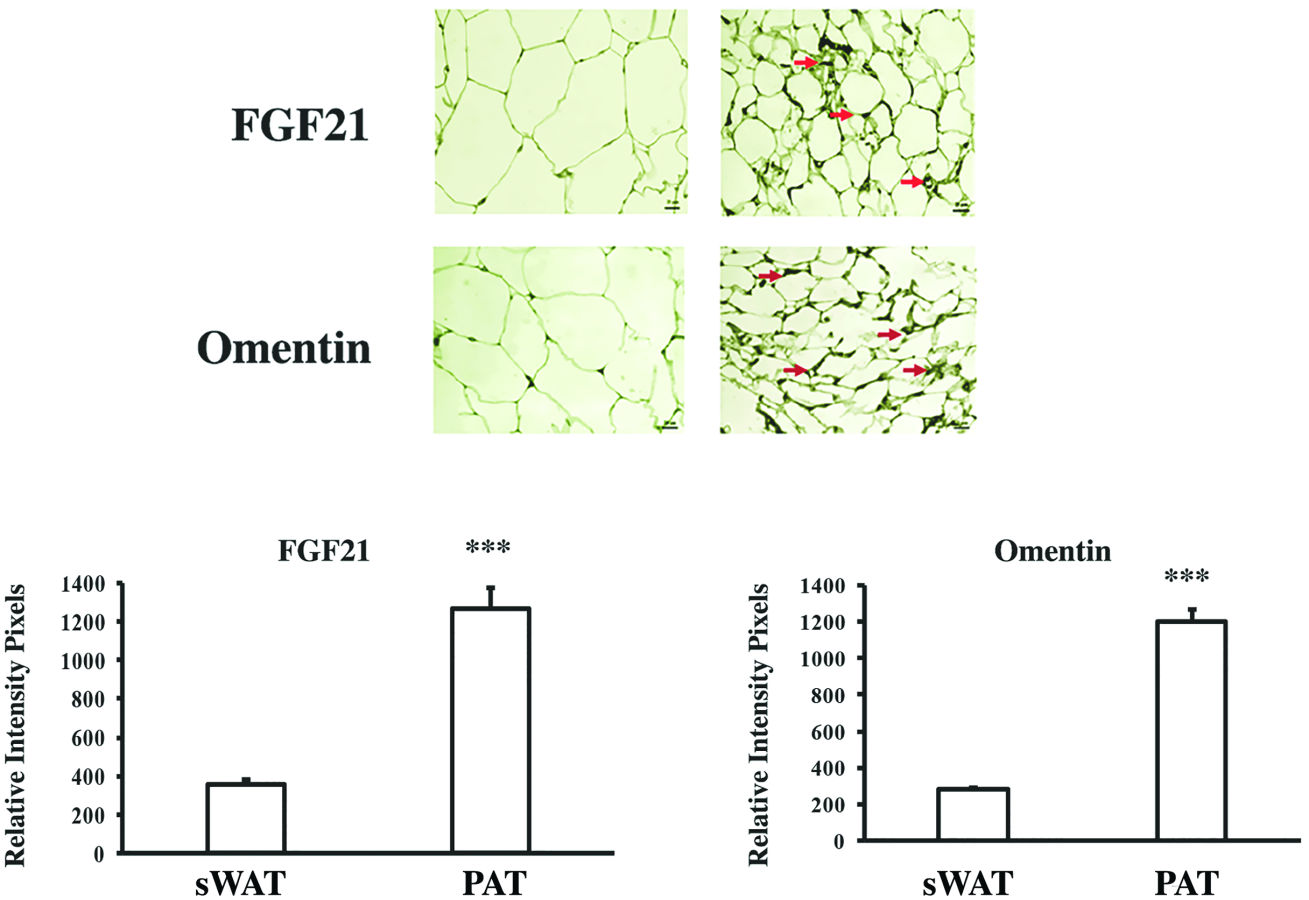
Expression of FGF21 and omentin in subcutaneous adipose tissue (sWAT) and periaortic adipose tissue. Representative image showing immunohistochemical staining for FGF21 and omentin (n = 8). A paired t-test was applied to determine significant differences between averages. (***) p<0.001 indicates a statistically significant difference in the levels of FGF21 and omentin expression between sWAT and PAT.

To identify molecules that could have a direct effect on vascular metabolism, the expression of omentin and FGF21 was evaluated by immunohistochemistry in the eight-paired sWAT and PAT samples previously used in morphological studies, Fig 1. Both FGF21 and omentin were expressed much more in PAT than in sWAT, Fig 4.

### Expression of thermogenic proteins in PAT

**Fig 5:**
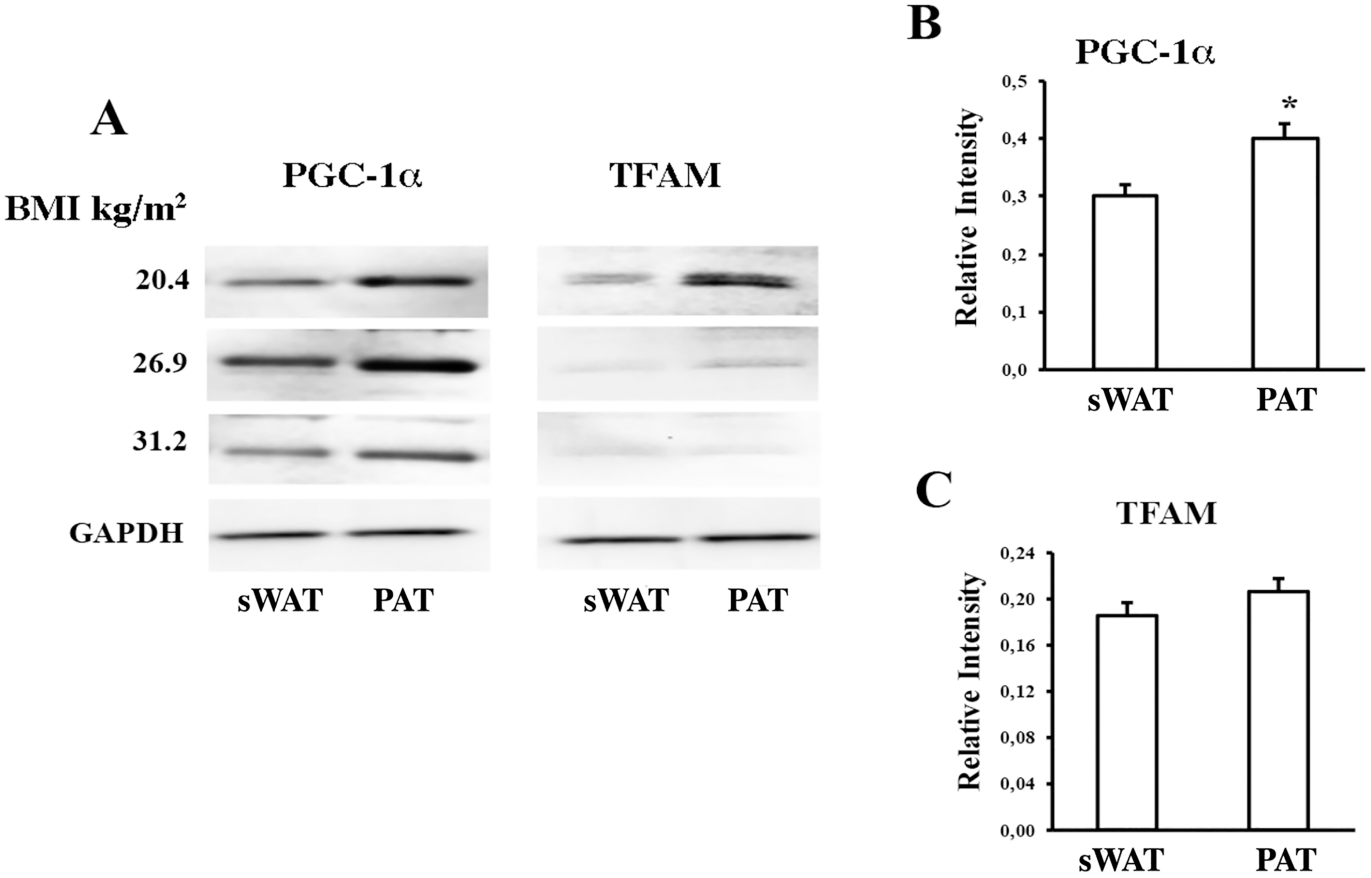
Thermogenic proteins in periaortic adipose tissue. (A) Representative western blots showing PPARg coactivator 1 alpha (PGC-1a) and transcription factor A, mitochondrial (TFAM) levels in total protein extracts from patients with normal (18–25 kg/m^2^), overweight (25–30 kg/m^2^), and obese (>30 kg/m^2^) body mass index (BMI). Extracts were obtained from subcutaneous adipose tissue (sWAT) and PAT adipocyte precursor cells. (B and C) Quantification of relative intensity levels. Data are expressed as mean ± standard deviation (n = 26). * A paired t-test was applied to determine significant differences in the average expression of thermogenic proteins between sWAT and PAT Please see a previous comment.

Early results in mice indicated that adipose tissue surrounding the ascending aorta could be highly thermogenically active [12]. We thus evaluated adipocyte precursor cells from sWAT and PAT. These were isolated and studied in samples obtained from 26 patients, some of whom had a BMI within the obesity range. Total proteins were isolated and the expression of PGC-1a and TFAM as markers of thermogenesis and mitochondrial activity, respectively, was determined. As shown in Fig 5A, where representative results of patients with different levels of BMI are presented, PGC-1a levels were significantly higher in PAT than in sWAT, regardless of BMI, (p<0.05 for all patients) Fig 5B. TFAM exhibited a similar tendency towards higher expression in PAT than in sWAT Fig 5C.

### Thermogenic proteins in PAT and their relationship with body Fat

**Fig 6:**
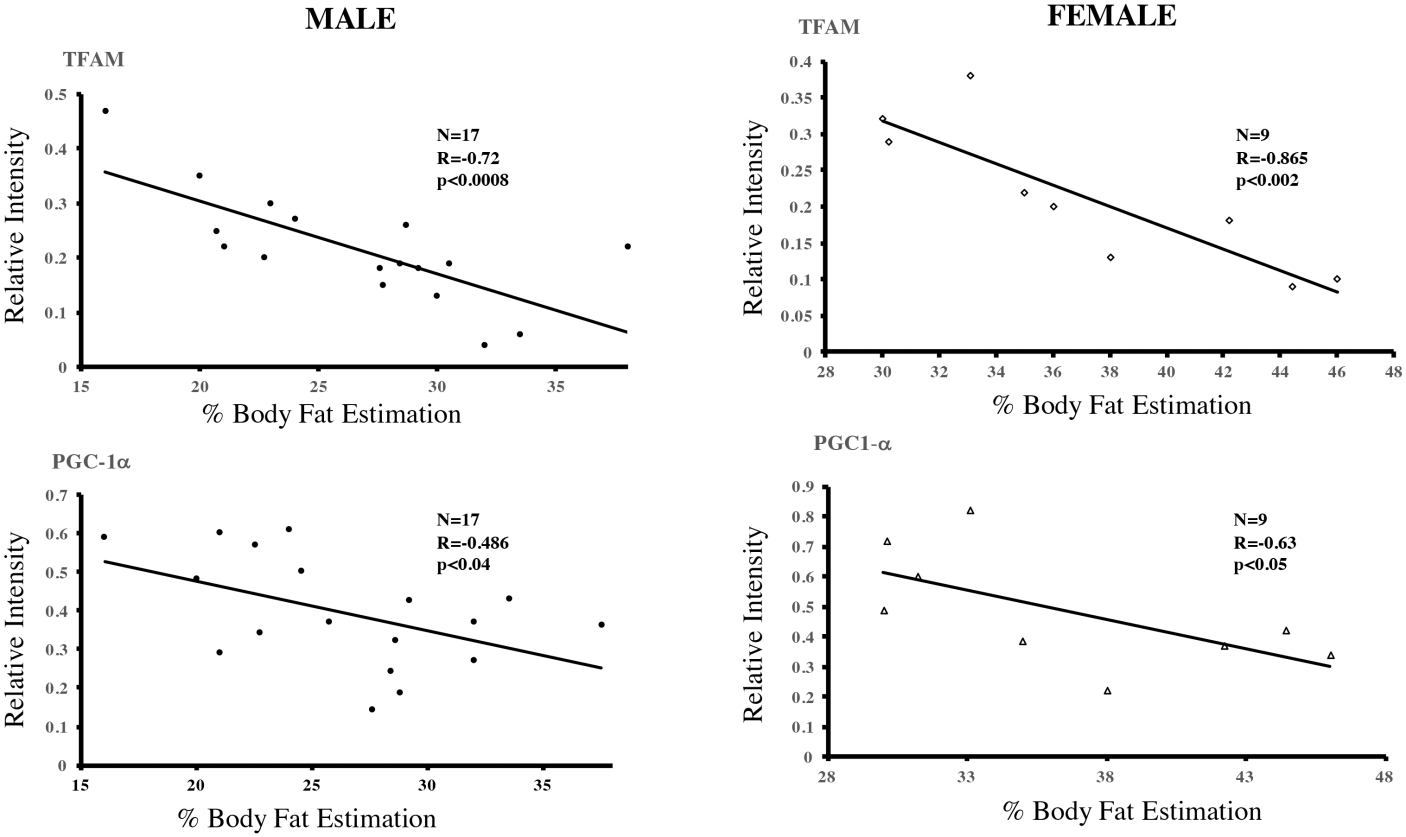
Expression of thermogenic proteins in periaortic adipose tissue (PAT) and correlation with body mass index (BMI). Based on the data in Fig 5, and the clinical data of patients a Pearson’s correlation analysis between the expression of thermogenic proteins and Body fat % estimation was carried out. Body Fat estimation was calculated by CUN-BAE method [19]. PPARg coactivator 1 alpha (PGC-1a) in male (r = -0.48 p<0.04) and female (r = -0.63, p<0.05) and TFAM in male (r = -0.72, p<0.0008) and female (r = -0.86, p<0.002) decreased significantly with progressive weight gain.

The different expression of the thermogenic proteins analyzed in sWAT and PAT samples led us to study in detail the clinical conditions of the patients [4]. No correlation was found between these proteins’ expression in the two tissue types and diabetes or lipid alterations. However, when assessing the relationship between protein expression of PGC-1a in male (r = -0.48 p<0.04) and female (r = -0.61, p<0.05) and TFAM in male (r = -0.72, p<0.0008) and female (r = -0.86, p<0.002). and Body Fat % estimation (CUN-BAE) using a Pearson correlation test, an inverse correlation was observed in the 26 patients analyzed Fig 6. This appears to suggest that weight gain may determine a morpho-physiological change in periaortic adipocytes, causing them to lose their thermogenic character and marking a possible increase in the risk of cardiovascular disease.

## Discussion

In recent years, metabolic studies sought to characterize the local function of adipose tissue surrounding blood vessels and its relation to cardiovascular metabolism. This has led to the discovery that besides secreting different adipokines to influence vascular metabolism, PVAT of mesenteric arteries, coronary arteries, and the aorta have different phenotypes caused by white and brown-like adipocytes [22, 23]. However, information about energy metabolism and endocrine or paracrine activity of the adipose tissue surrounding the ascending aorta in humans has remained scarce. Studies carried out on periaortic fat have been performed using imaging models [4, 24]. In these, the relationship between an increase in body weight and periaortic adipose tissue has been highlighted, including for its potential use as a marker of the risk of cardiovascular disease. In the present study, we present new information on the expression of proteins involved in thermogenesis in human samples of PAT. Moreover, we report for the first-time results that relate changes in some thermogenic protein of PAT with a clinical condition such as obesity. The adipose cells were evaluated after differentiation form precursor ADMSC. In addition, a histological examination of different samples of sWAT and PAT reported significant differences in adipocyte volume density Fig 1A and 1B. PAT samples showed high levels of the adipokines FGF21 and omentin when compared to sWAT, suggesting that PAT might play a role in adjusting vascular tone.

Previous studies have reported that in an obese state, adipose cells are hypertrophic, thus increasing the risk of cardiovascular disease [25]. However, adipocyte size differs according to anatomical location and partly determines energetic and metabolic functions [26, 27]. We report that adipocytes were significantly smaller in PAT compared to sWAT. Interestingly, correlation analyses showed that the difference between the two adipose tissue locations decreased significantly with increasing BMI. This suggests that although PAT adipocytes are smaller and more energetically active, progressive weight gain may change their beneficial phenotype. This observation may reflect results from mouse studies, whereby significant weight loss was seen to induce favorable morphological changes in PVAT adipocytes, including a reduced size and improved function [28, 29].

The activation of thermogenesis and energy expenditure are thought to be related to metabolic changes, because obese or overweight people have low thermogenic activity [30, 31]. Recent reports show that PVAT presents a BAT phenotype, which might protect against atherosclerosis and prevent the accumulation of lipids and inflammation in the vascular wall. To this end, mice subjected to high diets in thermoneutral conditions develop atherosclerosis, whereas cold-induced mice activate thermogenesis, which protects against vascular damage even under a high-calory diet [12, 32]. Our results indicate that, compared to sWAT, PAT causes significant expression of proteins involved in thermogenesis such as PGC-1a, in which significantly correlation was observed when analyzing data according to patients’ Body fat percentage. Seemingly, TFAM tended to be more expressed in PAT, although this was not significant. Interestingly, correlation analyses between the expression of TFAM and Body fat % indicated that the levels of TFAM were inversely proportional to BMI, Fig 5B. Although the number of patients was limited, the increase of post-operative glycemic control in some patients without previous diagnostic of diabetes, probably affect a possible influence in the characteristic of periaortic adipose tissue. These observations allow us to hypothesize that PAT is related to a thermogenically more active phenotype, which could be modulated at several levels in adverse conditions such as obesity. First, at a morphological level, adipocytes are smaller in PAT than in sWAT, which may be associated with a dynamic use of triglycerides as fuel to activate energy expenditure, thus reflecting a BAT-like phenotype [33]. Second, proteins such as PGC-1a are highly expressed in PAT, which, in principle, could protect PAT against atherosclerosis. Third, obesity might cause changes in the expression of proteins that promote mitochondrial activity and energy expenditure in blood vessels. This would result in expanded adipose tissue, which is related to cardiovascular risk [24]. Unlike individuals without detectable BAT, those with sufficient BAT present less total cholesterol and LDL [30]. We performed an evaluation of UCP-1 levels in all patients, however the expression of this protein in PAT was inconclusive. It is possible that the energetic function of the PAT cells may be independent of UCP-1, as has been demonstrated in some Beige fat cells. Subsequent studies should functionally assess the thermogenic capacity of PAT and the possible association with creatine-driven substrate cycle or Ca^2^ cycling [34, 35].

Another important challenge in understanding the functional role of adipose tissue based on its anatomical location is to investigate adipokines expression and their metabolic effect. Based on histological studies, we found that PAT expressed high levels of FGF21 and omentin compared to sWAT. These findings confirm that PAT might have functions related to vascular metabolism. This is in line with studies on Wistar rats showing that FGF21 increases energy expenditure and dramatically improves atherosclerosis conditions by decreasing LDL levels and increasing HDL [36–38]. Omentin is an anti-inflammatory adipokine with a cardioprotective effect that plays an important role in vasodilatation [39].

It should be noted that the study suffered from some limitations. The amount of tissue of sWAT and PAT origin obtained from operated patients was limited, making it difficult to perform all molecular and morphological studies in all patients. Thus, it is important to increase the number of patients to achieve a more realistic clinical approach. Additionally, analysis of other adipokines could give a better functional understanding of the local effects of PAT.

In conclusion, we suggest that PAT has a thermogenic phenotype, which grants it a protective character. The benefits of this type of adipose tissue might be lost with progressive weight gain. These data offer an interesting overview of periaortic adipose tissue metabolism and its potential use as a marker of cardiovascular disease risk. Further studies will be required to dynamically evaluate the activity of this adipose tissue in different clinical conditions and to determine whether weight reduction reestablishes endocrine and paracrine thermogenic benefits.
